# Evaluation of Radioiodinated Fluoronicotinamide/Fluoropicolinamide-Benzamide Derivatives as Theranostic Agents for Melanoma

**DOI:** 10.3390/ijms21186597

**Published:** 2020-09-09

**Authors:** Chao-Cheng Chen, Yang-Yi Chen, Yi-Hsuan Lo, Ming-Hsien Lin, Chih-Hsien Chang, Chuan-Lin Chen, Hsin-Ell Wang, Chun-Yi Wu

**Affiliations:** 1 Department of Biomedical Imaging and Radiological Sciences, National Yang-Ming University, Taipei 112, Taiwan; jimmy10421@hotmail.com (C.-C.C.); youth910766@gmail.com (Y.-Y.C.); aaa8579@hotmail.com (Y.-H.L.); chchang@iner.gov.tw (C.-H.C.); clchen2@ym.edu.tw (C.-L.C.); hewang@ym.edu.tw (H.-E.W.); 2 Department of Nuclear Medicine, Taipei City Hospital Zhongxiao Branch, Taipei 115, Taiwan; cha108009@chgh.org.tw; 3 Department of Nuclear Medicine, Cheng Hsin General Hospital, Taipei 112, Taiwan; 4 Institute of Nuclear Energy Research, Taoyuan 325, Taiwan

**Keywords:** melanoma, ^131^I-iodofluoropicolinamide benzamide (^131^I-IFPABZA), ^131^I-iodofluoronicotiamide benzamide (^131^I-IFNABZA), theranostic agent

## Abstract

Malignant melanoma is the most harmful type of skin cancer and its incidence has increased in this past decade. Early diagnosis and treatment are urgently desired. In this study, we conjugated picolinamide/nicotinamide with the pharmacophore of ^131^I-MIP-1145 to develop ^131^I-iodofluoropicolinamide benzamide (^131^I-IFPABZA) and ^131^I-iodofluoronicotiamide benzamide (^131^I-IFNABZA) with acceptable radiochemical yield (40 ± 5%) and high radiochemical purity (>98%). We also presented their biological characteristics in melanoma-bearing mouse models. ^131^I-IFPABZA (Log P = 2.01) was more lipophilic than ^131^I-IFNABZA (Log P = 1.49). B16F10-bearing mice injected with ^131^I-IFNABZA exhibited higher tumor-to-muscle ratio (*T/M*) than those administered with ^131^I-IFPABZA in planar γ-imaging and biodistribution studies. However, the imaging of ^131^I-IFNABZA- and ^131^I-IFPABZA-injected mice only showed marginal tumor uptake in A375 amelanotic melanoma-bearing mice throughout the experiment period, indicating the high binding affinity of these two radiotracers to melanin. Comparing the radiation-absorbed dose of ^131^I-IFNABZA with the melanin-targeted agents reported in the literature, ^131^I-IFNABZA exerts lower doses to normal tissues on the basis of similar tumor dose. Based on the in vitro and in vivo studies, we clearly demonstrated the potential of using ^131^I-IFNABZA as a theranostic agent against melanoma.

## 1. Introduction

Among skin cancers, malignant melanoma is the deadliest form and results in the majority of skin cancer deaths around the world [1,2]. The annual incidence of malignant melanoma has risen rapidly, especially in North America, owing to genetic defects and increased ultraviolet light exposure caused by global environmental change and destruction of the ozonosphere [3]. After surgery, the 5-year survival rate is >90% in patients with localized and noninvasive melanoma [4]. Once metastasized, melanoma becomes uncontrollable, and the survival rate dramatically decreases to less than 20% due to limited therapeutic options [5,6].

Melanin is composed of two biogenetically related pigments, eumelanin and pheomelanin [7]. The primary function of melanin is to protect skin against UV-induced damage. Most of the malignant melanoma cells overexpressed melanin because of increased tyrosinase activity [8,9]. Therefore, melanin is thought to be a potential target for early detection and treatment for advanced malignant melanoma. Michelot et al. first indicated a significant accumulation of ^125^I-iodobenzamide (^125^I-IBZA) in B16 murine melanoma (6.75%ID/g) and liver (6.04%ID/g) at 1 h post injection [10,11]. In 2010, Joyal et al. discovered a superior melanin-targeting ability of an iodine-123/131 labeled fluorobenzoate-benzamide derivative (^123/131^I-MIP-1145) [12]. The uptake of ^131^I-MIP-1145 in melanin-expressed SK-MEL-3 human melanoma xenograft (8.82 ± 3.55%ID/g) was significantly higher than that in the amelanotic A375 one (1.19 ± 0.07%ID/g) at 4 h after intravenous injection [12]. The growth inhibition effect on the SK-MEL-3 xenograft was also noticed when the tumor-bearing mice received ^131^I-MIP-1145 administration (1 × 25, 2 × 25, and 3 × 25 MBq) [12]. However, the high lipophilicity of ^123/131^I-MIP-1145 (logP = 4.5) led to apparent radioactivity accumulation in liver and gastrointestinal tract and resulted in high radiation absorption dose in normal tissues [12]. Several studies reported that nicotinamide (^18^F-MEL050) [13,14] and picolinamide derivatives (^18^F-5-FPN) [15] showed a prolonged retention in melanotic melanoma and elevated clearance rate in normal tissues when compared with benzamides analogues. In our previous study, we successfully conjugated an ^131^I-labeled benzamide derivative with a nicotinamide to give ^131^I-ICNA and demonstrated its potency in remarkable melanoma targeting along with low retention in normal tissues [16].

Based on the previous results of ^131^I-MIP-1145 and ^131^I-ICNA, we aim to conjugate picolinamide and nicotinamide with the pharmacophore of ^131^I-MIP-1145 to determine the feasibility of ^131^I-iodofluoropicolinamide benzamide (^131^I-IFPABZA) and ^131^I-iodofluoronicotiamide benzamide (^131^I-IFNABZA) as a theranostic agent to target melanin-expressed melanoma in this study. To the best of our knowledge, this study should be the first one to investigate the biological characteristics of fluoropicolinamide- and fluoronicotinamide-benzamide derivatives and to evaluate their clinical potential as a theranostic agent against melanoma.

## 2. Results

### 2.1. The Preparation of ^131^I-IFPABZA and ^131^I-IFNABZA

**Figure 1:**
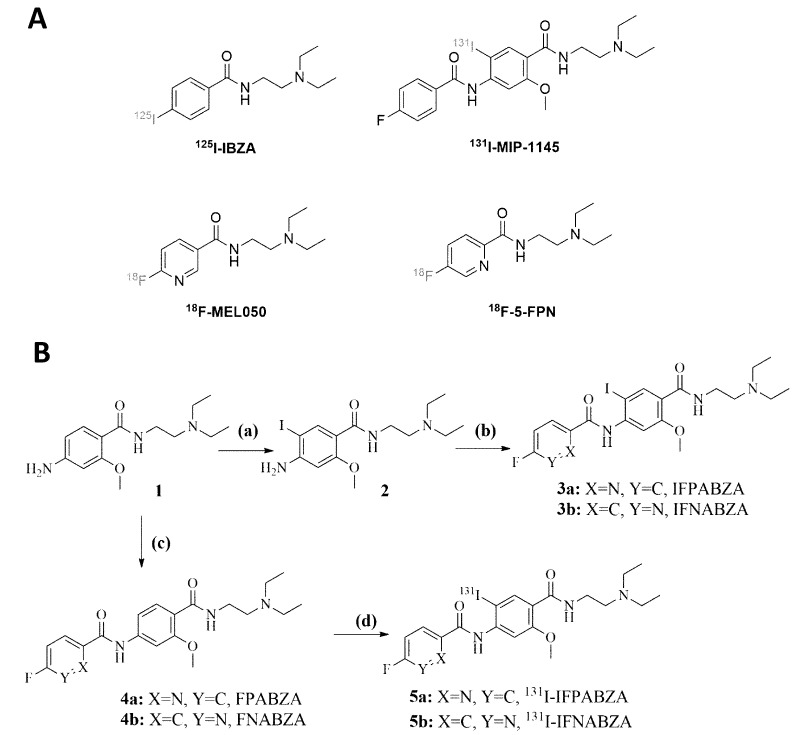
(**A**) The previously reported radiotracers for melanoma. (**B**) Synthetic scheme of non-radioactive ioflouropicolinamide-benzamide/iofluronicotinamide-benzamide (3a/3b), precursor (4a/4b), and ^131^I-ioflouropicolinamide-benzamide/iofluronicotinamide-benzamide (5a/5b). Reaction conditions: (**a**) CaCO_3_, and ICl, in MeOH/H_2_O, ambient temperature for 10 h; (**b**) EDC, HOBt, DIPEA, DEDA, and 5-fluoropyridine-2-carboxylic acid/6-fluoronicotinic acid, in anhydrous THF, ambient temperature for 24 h; (**c**) 5-fluoropyridine-2-carboxylic acid/6-fluoronicotinic acid, thionyl chloride, and K_2_CO_3_, in anhydrous THF, ambient temperature for 16 h; (**d**) Tl(TFA)_3_, and ^131^I-NaI solution in 0.5% NaHCO_3_ solution (pH 8.6~8.8), ambient temperature for 10 min.

**Figure 2:**
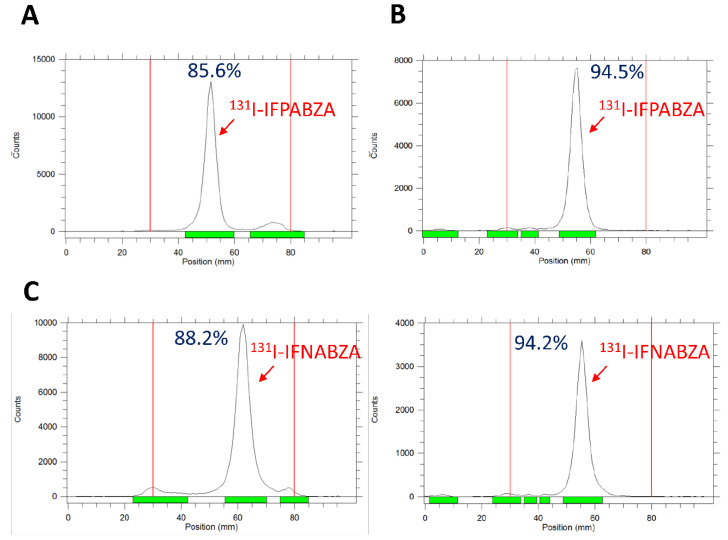
(**A**,**C**) Radio thin-layer chromatography analysis of the reaction mixture and (**B**,**D**) the purified ^131^I-IFPABZA and ^131^I-IFNABZA. The radiolabeling efficiency and the radiochemical purity was determined by radio-TLC using MeOH/CH_2_Cl_2_ = 1:10 (*v*/*v*) and 1:8 (*v*/*v*) as eluting agent for ^131^I-IFPABZA and ^131^I-IFNABZA, respectively.

The synthetic scheme of non-radioactive ioflouropicolinamide-benzamide (IFPABZA), iofluronicotinamide-benzamide (IFNABZA), and the precursors for radiolabeling is illustrated in Figure 1. The I-131 was introduced to compound 4a and 4b by the radioiododethallation method. The labeling efficiency of ^131^I-IFPABZA and ^131^I-IFNABZA was around 85 ± 5% (Figure 2A,C). After purification, the radiochemical purity of both compounds was greater than 90% (Figure 2B,D). The radiochemical yield of ^131^I-IFPABZA and ^131^I-IFNABZA was approximately 40 ± 5% and the total preparation time for both compounds was about 45 min.

### 2.2. Partition Coefficient and In Vitro Stability of ^131^I-IFPABZA and ^131^I-IFNABZA

**Figure 3:**
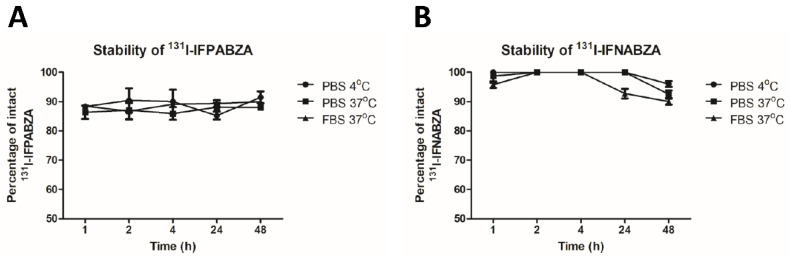
In vitro stability of (**A**) ^131^I-IFPABZA and (**B**) ^131^I-IFNABZA in either PBS or fetal bovine serum (FBS).

The determined log P value of ^131^I-IFPABZA and ^131^I-IFNABZA was 2.01 ± 0.12 and 1.19 ± 0.07, respectively, indicating both of them were considerably hydrophobic compounds. In the stability tests, the percentage of intact ^131^I-IFNABZA was 92.8 ± 1.6% after a 24 h incubation in FBS at 37 °C, but that of ^131^I-IFPABZA was comparatively lower (89.4 ± 1.1%) (Figure 3A,B). In fact, at the first time point, the percentage of intact ^131^I-IFNABZA incubated in PBS was below 90% at either 4 or 37 °C (Figure 3A), suggesting a relatively poor stability.

### 2.3. In Vitro Binding of ^131^I-IFPABZA and ^131^I-IFNABZA to Melanin

**Figure 4:**
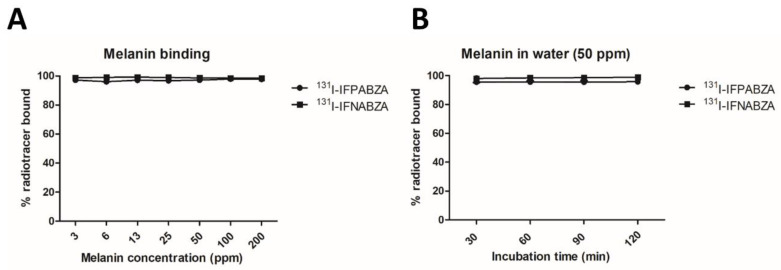
In vitro binding assay between ^131^I-IFPABZA/^131^I-IFNABZA and melanin. (**A**) Binding with various concentrations of melanin at 37 °C for 1 h. (**B**) Bound ratio of radiotracers with 50 ppm of melanin suspension at 37 °C after different incubation time.

Both ^131^I-IFPABZA and ^131^I-IFNABZA showed a high binding affinity to melanin. More than 98% radioactivity of ^131^I-IFPABZA and ^131^I-IFNABZA bound to melanin at a concentration ranging from 3 to 200 ppm at 1 h post incubation at 37 °C (Figure 4A). At a concentration of 50 ppm melanin suspension, the bound percentage of these two compounds rapidly reached 98% after initial 30 min incubation and remained high for 2 h (Figure 4B).

### 2.4. Assessment of In Vitro Cellular Uptake

**Figure 5:**
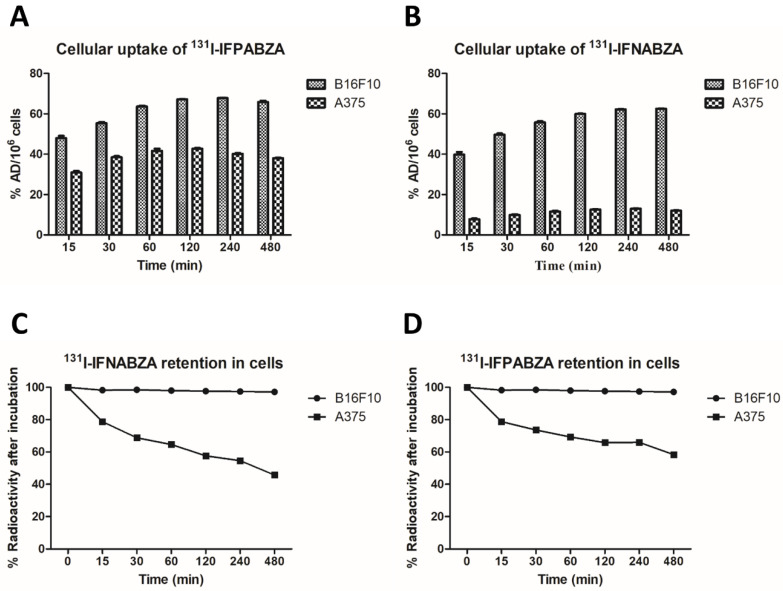
Cellular uptake of (**A**) ^131^I-IFPABZA and (**B**) ^131^I-IFNABZA in B16F10 murine melanoma and A375 human melanoma cells. Washout studies of (**C**) ^131^I-IFPABZA and (**D**) ^131^I-IFNABZA in B16F10 and A375 cells All treatments were repeated in six times (n = 6), and data were expressed as mean ± SD.

The cellular uptake of ^131^I-IFPABZA and ^131^I-IFNABZA (expressed as %AD/10^6^ cells) increased with time, reaching a maximum accumulation of 67.8 ± 0.2%AD/10^6^ cells at 240 min post incubation, and 62.6 ± 0.2%AD/10^6^ cells at 480 min post incubation, respectively, and remained high throughout the 8 h study period (Figure 5A,B). However, the accumulation of both radiotracers in A375 cells was significantly lower than that in B16F10 cells. The peak uptake of ^131^I-IFPABZA in A375 cells was 42.8 ± 0.3%AD/10^6^ cells (at 120 min post incubation), while that of ^131^I-IFNABZA was below 15%AD/10^6^ cells at all time points. ^131^I-IFNABZA possessed a noted higher melanotic-to-amelanotic ratio than ^131^I-IFPABZA.

The washout studies showed that the efflux out of the B16F10 of ^131^I-IFPABZA and ^131^I-IFNABZA was not obviously even at 8 h post incubation with fresh medium, implying their high specificity toward melanin (Figure 5C,D). The percentage of these two drugs retained in B16F10 cells remained >95% throughout the experiment period. However, when incubated with fresh medium, the accumulation of ^131^I-IFPABZA and ^131^I-IFNABZA in A375 cells decreased with time, and the intracellular radioactivity percentage reached 44.2 ± 0.3 and 34.0 ± 0.4%AD/10^6^ cells, respectively, suggesting the majority of their cellular uptake in A375 cells accounted for non-specific binding.

### 2.5. Scintigraphic Imaging

**Figure 6:**
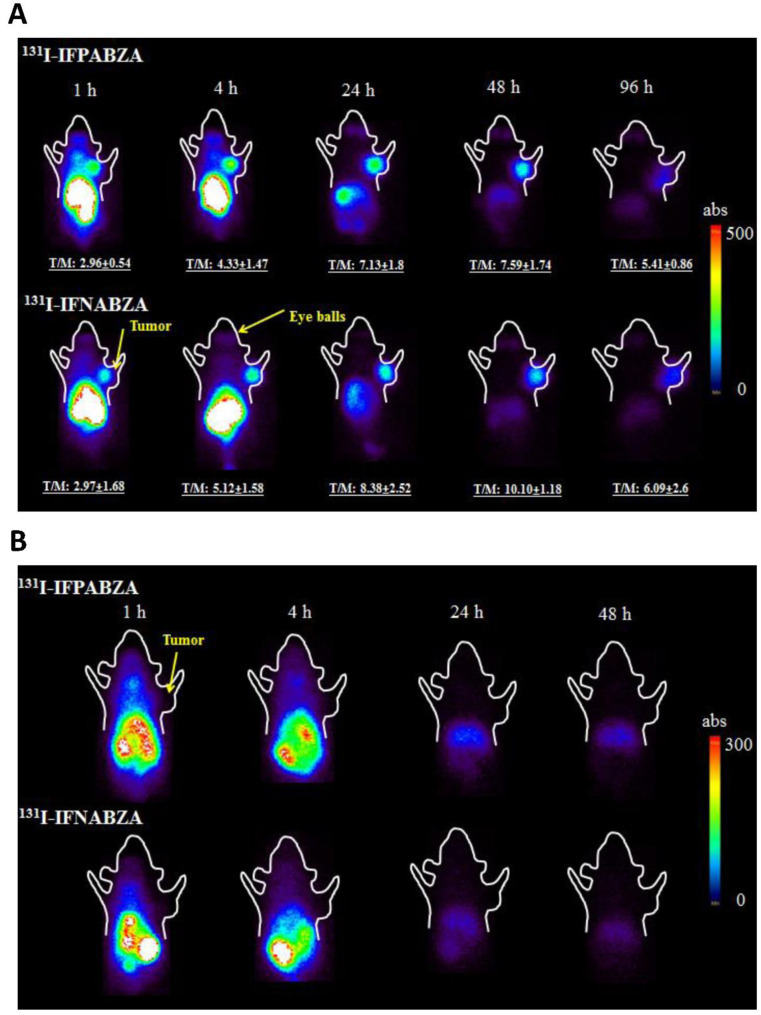
Representative coronal scintigraphic images of (**A**) B16F10 tumor-bearing C57BL/c mice at 1, 4, 24, 48, and 96 h p.i. (**B**) A375 amelanotic xenograft-bearing BALB/c nude mice at 1, 4, 24, and 48 h administered with ^131^I-IFPABZA (top) and ^131^I-IFNABZA (bottom).

The scintigraphic imaging showed that both ^131^I-IFPABZA and ^131^I-IFNABZA were retained in B16F10 tumors (Figure 6A), but not in A375 xenografts (Figure 6B). The T/M in ^131^I-IFPABZA-injected B16F10-bearing mice increased from 2.96 ± 0.54 at 1 h p.i. to 7.59 ± 1.76 at 48 h p.i., while that of ^131^I-IFNABZA increased from 2.97 ± 1.68 to 10.10 ± 1.18. At 96 h post administration, the T/M of ^131^I-IFPABZA and ^131^I-IFNABZA in B16F10-bearing mice was 5.41 ± 0.86 and 6.09 ± 2.60, respectively. In contrast to B16F10 melanoma, the results of the scintigraphic imaging demonstrated that the retention of ^131^I-IFPABZA and ^131^I-IFNABZA was only negligible in tumor throughout the experiment period. These results suggested that both ^131^I-IFPBZA and ^131^I-IFNABZA exhibited superior in vivo melanin targeting ability. Except for tumor, high radioactivity accumulation in the abdomen was also noticed at 1 h p.i., but most of the radioactivity of the two radiotracers retained in the abdomen was washed out at 24 h p.i. The clearance rate of ^131^I-IFNABZA was slightly higher than that of ^131^I-IFPBZA.

### 2.6. Biodistribution Study

Considering the binding affinity and specificity determined by in vitro and scintigraphic imaging, we only performed the biodistribution studies of ^131^I-IFNABZA in B16F10 melanoma- and A375 amelanotic melanoma-bearing mice (Table 1 and Table S1). At 5 min p.i., high radioactivity retention was noticed in lung (23.49 ± 2.20%ID/g), kidneys (26.80 ± 2.32%ID/g), and liver (11.91 ± 0.93%ID/g), accompanied with low radioactivity in blood (1.70 ± 0.15%ID/g), suggesting that a fast distribution of ^131^I-IFNABZA to the organs from the blood (Table 1). Although noted uptake in normal organs at the initial time points was observed, most of the radioactivity in normal organs was rapidly washed out and eliminated through urinary and intestinal routes within 24 h, which was consistent with those noticed in the scintigraphic images. A rapid and significant tumor accumulation of ^131^I-IFNABZA at 5 min p.i. (4.29 ± 0.93%ID/g) was observed and the tumor uptake was 5.84 ± 1.80, 5.19 ± 2.84, 5.06 ± 2.09, 5.17 ± 1.53, and 1.51 ± 0.34%ID/g at 1, 4, 24, 48, and 96 h p.i., respectively, in B16F10 melanoma-bearing mice. The T/M was 1.48 ± 0.33, 9.90 ± 3.66, 34.60 ± 19.49, 168.70 ± 89.52, and 258.50 ± 76.50, at 5 min, 1, 4, 24, 48 h p.i., respectively, and then, declined to 151.0 ± 34.0 at 96 h p.i. The apparent uptake in black eyeballs, another tissue with a high amount of melanin in C57BL/6 mice, was also noticed (Table 1). The uptake in eyeballs and A375 tumors was 1.20 ± 0.26 and 1.66 ± 0.39%ID/g at 5 min p.i., respectively, and decreased to 0.04 ± 0.01 and 0.10 ± 0.05 at 24 h p.i., respectively (Table S1). The distribution of ^131^I-IFNABZA in A375 amelanotic melanoma-bearing mice was similar to those observed in B16F10 melanoma-bearing mice, except for low tumor and eyeball uptake. High accumulation of ^131^I-IFNABZA in lung, kidney, and liver was also noticed at initial time points, and the radioactivity rapidly eliminated within 24 h (Table S1).

### 2.7. Estimated Absorption Dose Calculation

The results of the biodistribution study were further applied to calculate the estimated absorption dose in humans. As shown in Table 2, liver exhibited the highest absorbed dose (0.122 mSv/MBq), followed by spleen (0.151 mSv/MBq). Even though high accumulation of ^131^I-IFNABZA in lung was noticed at initial time points, the estimated absorbed dose was only 2.25 × 10^−2^ mSv/MBq. Overall, the estimated effective dose for the whole body in a 73 kg adult human was 3.02 × 10^−2^ mSv/MBq.

## 3. Discussion

Several radioactive melanin-targeting probes have been developed for the diagnosis and treatment of melanoma. The drugs that received the most attention were benzamide analogues. Bonnot-Duquennoy et al. found that two doses of ^131^I-ICF01012 injection (18.5 × 2 MBq) can extend the tumor doubling time from 2.41 to 5.97 d, but only slightly improved the survival time from 32 to 39 d [17]. Joyal et al. also showed that tumor growth inhibition was significant after three doses of ^131^I-MIP-1145 administration (25 × 3 MBq) [12]. Unfortunately, the high accumulation in the gastrointestinal tract raised a concern about normal tissue injury. Xu et al. developed ^131^I-5-IPN as a therapeutic agent against murine B16F10 melanoma. However, its therapeutic efficacy was not noted, even though the tumor uptake reached 16.37%ID/g at 1 h p.i. [18]. In the present study, we prepared a radioiodine-labeled fluoropicolinamide-benzamide derivative (^131^I-IFPABZA) and fluoronicotinamide-benzamide derivative (^131^I-IFNABZA) and determined their in vitro and in vivo biological characteristics. The radioiodination of aromatic compound (^131^I-IFPABZA and ^131^I-IFNABZA) was performed through radioiododothallation instead of the electrophilic substitution mechanism because the amide linkage, an electron withdrawing group, is located in the *ortho* position on the aromatic ring [12,19,20].

A series of in vitro melanin-binding studies revealed that both ^131^I-IFPABZA and ^131^I-IFNABZA could rapidly and strongly bind to melanin (Figure 3). In the cellular uptake studies, the accumulation of ^131^I-IFNABZA in melanotic B16F10 cells was 4.6-fold higher than that in amelanotic A375 cells after 2 h incubation, while the ratio was only 1.6 for ^131^I-IFPABZA (Figure 4). Although the scintigraphic imaging of B16F10 melanoma-bearing mice clearly showed a significant accumulation and prolonged retention of ^131^I-IFPABZA and ^131^I-IFNABZA in the tumor region, the *T/M* of ^131^I-IFNABZA at 48 h p.i. (10.10 ± 1.18) was superior to that of ^131^I-IFPABZA (7.59 ± 1.74) (Figure 5). Taken together, our results suggested that ^131^I-IFNABZA should be a potential melanin-targeting agent rather than ^131^I-IFPABZA.

Compared with B16F10 melanoma, biodistribution studies clearly revealed that ^131^I-IFNABZA was rarely found in A375 amelanotic melanoma, which was consistent with the results derived from cellular uptake studies and scintigraphic imaging. Except for melanotic tumor, we noticed the lung uptake at 5 and 15 min p.i. was 23.49 ± 2.20 and 13.87 ± 2.64%ID/g, respectively, which were significantly higher than blood activity at the same time points. In order to rule out that this was caused by the precipitation in lung, we assessed the experiments of ^131^I-IFNABZA dissolved in various excipients (Table S2), and found there was no apparent difference in radioactivity accumulated in lung (ranging from 14 to 16%ID/g) and other organs, suggesting the precipitation that originated from relatively poor solubility was not the reason for unexpected lung retention. Another possible explanation is that ^131^I-IFNABZA may specifically bind to the sigma receptor, which is highly expressed in lung. In fact, previous literature reported that benzamide derivatives showed high-affinity binding to the sigma receptor [21,22].

We summarized the lipophilicity and the results of the biodistribution study of numerous published melanin-targeting agents, including benzamide, nicotinamide, and picolinamide analogues [10,14,15,16,18,23,24,25,26,27,28,29]. As illustrated in Figure S1A, melanin-targeting agents with high lipophilicity tended to exhibit high liver uptake rather than tumor uptake. Agents with a logP ranging from −1 to 1 showed a tendency to be localized in tumor (Figure S1B). In our study, the accumulated radioactivity in B16F10 tumors kept at around 5%ID/g till 48 h p.i., and decreased to 1.51 ± 0.34 at 96 h p.i. A prolonged retention of the fluoronicotinamide-benzamide conjugate in B16F10 melanoma was noticed as compared to previous benzamide derivatives, which was consistent with the report by Greguric et al. and Liu et al. [14,15]. At 48 h p.i., the *T/M* increased to 258.5 ± 76.50, which was much higher than ^131^I-Iochlonicotinamide (*T/M* = 107.5) [16] and ^131^I-5-IPN (*T/M* = 121.5) [18].

To evaluate the potential of ^131^I-IFNABZA in melanoma treatment, dosimetry was estimated by OLINDA software. The low absorbed dose in the normal organ was attributed to the fast elimination rate of ^131^I-IFNABZA. The estimated absorbed dose of liver was 0.112 mSv/MBq, which was similar to that receiving ^131^I-MIP-1145 (0.086 mSv/MBq) and ^131^I-ICNA (0.13 mSv/MBq) injection, but the estimated effective dose for the whole body was 46.3-fold and 1.5-fold lower than that of ^131^I-MIP-1145 and ^131^I-ICNA, respectively.

## 4. Materials and Methods

### 4.1. Reagents and Instruments

All reagents and solvents were purchased from commercial suppliers and used without further purification. 4-Amino-2-methoxybenzoic acid (97%), N,N-diisopropylethylamine (DIPEA, >98%), N,N-diethylethylenediamine (DEDA, >98%), ammonium hydroxide (NH_4_OH), iodine monochloride, and trifluoroacetic acid (TFA, 99%) were purchased from ACROS organics (Morris, NJ, USA). 1-Ethyl-3-(3-dimethylaminopropyl)carbodiimide (EDC) was purchased from Biosynth Carbosynth (Berkshire, UK). 1-Hydroxybenzotriazole hydrate (HOBt) and 6-fluoronicotinic acid (98%) were obtained from AK Scientific, Inc (Union City, CA, USA). 5-Fluoropyridine-2-carboxylic acid (98%) was obtained from Accela ChemBio Co., Ltd. (Shanghai, China). Magnesium sulfate (MgSO_4_), potassium carbonate (K_2_CO_3_), and thionyl chloride (SOCl_2_) were purchased from Merck KGaA (Darmstadt, Germany). ^131^I-NaI in 0.5% NaHCO_3_ solution (pH 8.6~8.8) was purchased from Institute of Isotopes Co., Ltd. (Budapest, Hungary). Thin layer chromatography (TLC) was performed on silica gel F254 aluminum-backed plates (Merck KgaA) with visualization under UV (254 nm). Nuclear magnetic resonance (NMR) spectra were recorded on the Bruker 400 UltraShield NMR spectrometer (Bruker) operating at 400 MHz for ^1^H NMR spectra and 100 MHz for ^13^C NMR spectra at the Instrumentation Resource Center of National Yang-Ming University (Taipei, Taiwan). The radioactivity was determined by the g-counter (PerkinElmer 1470 Automatic Gamma Scintillation, PerkinElmer Inc.)

### 4.2. Synthesis of ^131^I-IFPABZA and ^131^I-IFNABZA

#### 4.2.1. Synthesis of 4-amino-N-(2-(diethylamino)ethyl)-2-methoxybenzamide (**1**)

To a solution of 4-amino-2-methoxybenzoic acid (1.00 g, 5.98 mmol) in 25.0 mL of anhydrous THF, EDC•HCl (1.72 g, 8.97 mmol) and DIPEA (0.85 g, 6.58 mmol) were added. After stirring in an ice bath for 1 h, HOBt (1.01 g, 6.58 mmol) and DEDA (0.765 g, 6.58 mmol) were added to the reaction mixture. The solution was stirred at room temperature (r.t.) for 24 h and then, evaporated to dryness. The residue was redissolved with dichloromethane and then, washed with 4 N NaOH and brine twice. The collected organic layer was dried over MgSO_4_ and then concentrated. Column chromatography on silica gel eluting with methanol/dichloromethane (1/5, *v*/*v*) was used to afford the pure compound **1** (0.873 g, in 55% yield) as a brownish oil. ^1^H-NMR (CDCl_3_, 400MHz): δ 1.05 (t, J = 7.0 Hz, 6H), 2.57 (q, J = 6.8 Hz, 4H), 2.62 (t, J = 6.0 Hz, 2H), 3.49 (t, J = 5.2 Hz, 2H), 3.89 (s, 3H), 6.20 (s, 1H), 6.35 (d, J = 6.4 Hz, 1H), 8.03 (d, J = 8.4 Hz, 1H). ^13^C NMR (CDCl_3_): δ 12.1, 37.4, 46.7, 51.7, 55.5, 97.2, 107.5, 112.1, 133.9, 150.7, 159.2, 165.4.

#### 4.2.2. Synthesis of 4-amino-N-(2-(diethylamino)ethyl)-5-iodo-2-methoxybenzamide (**2**)

To a solution of compound **1** (1 g, 3.77 mmol) in 5.0 mL of methanol and 1.5 mL of H_2_O, CaCO_3_ (0.589 g, 5.88 mmol) and iodine monochloride solution in methanol (4.0 mmol, 2.5 mL) were added. The solvent was removed by rotary evaporator after stirring at r.t. for 10 h. The residue was redissolved in dichloromethane and then, washed with 4 N NaOH and brine twice. The collected organic layer was dried over MgSO_4_, and then concentrated. Column chromatography on silica gel eluting with methanol/dichloromethane (1/6, *v*/*v*) was used to afford the pure compound **2** (0.34 g, in 23% yield) as a yellowish solid. ^1^H-NMR (CDCl_3_, 400MHz): δ1.06 (t, J = 6.6 Hz, 6H), 2.61 (q, J = 6.7 Hz, 4H), 2.67 (t, J = 6.0 Hz, 2H), 3.50 (t, J = 4 Hz, 2H), 3.85 (s, 3H), 6.27 (s, 1H), 8.41 (s, 1H). ^13^C NMR (CDCl_3_): δ 11.6, 37.3, 47.0, 51.6, 55.8, 96.7, 113.7, 142.5, 150.6, 159.3, 164.3.

#### 4.2.3. Synthesis of N-(4-((2-(diethylamino)ethyl)carbamoyl)-2-iodo-5-methoxyphenyl)-5-fluoropico-linamide (**3a**) and N-(4-((2-(diethylamino)ethyl)carbamoyl)-2-iodo-5-methoxy-phenyl)-6-fluoronicotinamide (**3b**)

A solution of 5-fluoropyridine-2-carboxylic acid (60.3 mg, 0.427 mmol) in thionyl chloride (0.904 mL, 12.4 mmol) was reacted at 74 °C for 3 h. After the reaction, excess thionyl chloride was removed under reduced pressure. The residue was redissolved in anhydrous THF (2.0 mL). Compound **2** (0.21 g, 0.54 mmol) and potassium carbonate (114.7 mg, 0.83 mmol) were added to the mixture and allowed to stir at r.t. for 16 h. After evaporation, the residue was redissolved in dichloromethane, and washed with brine and ddH_2_O. The collected organic layer was dried over MgSO_4_, and then concentrated. The crude product was purified by column chromatography on silica gel using MeOH/CH_2_Cl_2_ (1:10, *v*/*v*) as the mobile phase to afford compound **3a** (0.048 g, in 22% yield) as a yellowish solid. The compound **3b** was prepared with the same procedure described above, except for using 6-fluoronicotinic acid (60.2 mg, 0.427 mmol) as the starting material. The chemical yield of compound **3b** (0.029 g, yellowish solid) was around 13%. Compound **3a**: ^1^H-NMR (CDCl_3_, 400MHz): δ1.05 (t, J = 7 Hz, 6H), 2.61 (q, J = 6.8 Hz, 4H), 2.65 (t, J = 6.2 Hz, 2H), 3.52 (t, J = 4.8 Hz, 2H), 4.01 (s, 3H), 8.28 (t, J = 2.4 Hz, 1H), 8.34 (d, J = 4.4 Hz, 1H), 8.44 (s, 1H), 8.53 (d, J = 4.4 Hz, 1H), 8.62 (s, 1H). ^13^C-NMR (CDCl_3_, 400MHz): δ11.5, 37.3, 47.0, 51.5, 56.1, 78.0, 103.7, 118.9, 124.3, 124.4, 137.2, 141.7, 141.9, 145.7, 158.6, 161.0, 161.8, 163.6. Compound **3b**: ^1^H-NMR (CDCl_3_, 400MHz): δ1.06 (t, J = 7 Hz, 6H), 2.59 (q, J = 6.8 Hz, 4H), 2.66 (t, J = 6.2 Hz, 2H), 3.52 (t, J = 4.4 Hz, 2H), 4.00 (s, 3H), 7.10 (t, J = 8.4 Hz, 1H), 8.32 (s, 1H), 8.38 (d, J = 4.4 Hz, 1H), 8.60 (s, 1H), 8.86 (s, 1H). ^13^C NMR (CDCl_3_): δ11.9, 37.5, 46.8, 51.4, 56.2, 78.9, 104.4, 119.7, 128.21, 128.25, 140.1, 141.7, 147.3, 147.5, 158.6, 162.5, 163.3, 164.3, 166.7

#### 4.2.4. Synthesis of N-(4-((2-(diethylamino)ethyl)carbamoyl)-3-methoxyphenyl)-5-fluoropicolinamide (**4a**) and N-(4-((2-(diethylamino)ethyl)carbamoyl)-3-methoxy-phenyl)-5- fluoronicotinamide (**4b**)

A solution of 5-fluoropyridine-2-carboxylic acid (175.0 mg, 1.24 mmol) in 2.6 mL of thionyl chloride (36.0 mmol) was reacted at 74 °C for 3 h. After the reaction, excess thionyl chloride was removed under reduced pressure. The residue was dissolved in 5.0 mL of anhydrous THF. To the solution, potassium carbonate (334.5 g, 2.42 mmol) and compound **1** (0.393 g, 1.48 mmol) in 10 mL of anhydrous THF were added. After stirring at r.t. for 16 h, the reaction mixture was evaporated to dryness. The resulting residue was redissolved in dichloromethane, and washed with the brine, dried over MgSO_4_, and concentrated. The crude product was purified by column chromatography on silica gel using methanol/dichloromethane (1:6, *v*/*v*) as the mobile phase to afford compound **4a** (yellowish solid, 0.289 g, in 60% yield). The compound **4b** was prepared with the same procedure described above, except for using 6-fluoronicotinic acid (60.2 mg, 0.427 mmol) as the starting material (yellowish solid, 0.241 g, in 50% yield). Compound **4a**: ^1^H-NMR (CDCl_3_, 400MHz): δ1.05 (t, J = 6.6 Hz, 6H), 2.60 (q, J = 6.8 Hz, 4H), 2.66 (t, J = 6.2 Hz, 2H), 3.53 (t, J = 4.8 Hz, 2H), 4.00 (s, 3H), 7.03 (d, J = 8.4 Hz, 1H), 7.95 (s, 1H), 8.18 (d, J = 8.4 Hz, 1H), 8.29, (t, J = 8.0, 1H), 8.40 (d, J = 6.4, 1H), 8.42 (d, J = 10.8, 1H). ^13^C NMR (CDCl_3_): δ11.8, 37.4, 46.8, 51.5, 55.9, 102.5, 112.2, 111.7, 117.6, 123.7, 124.7, 132.9, 140.7, 141.3, 147.8, 149.3, 158.5, 161.6, 164.85. Compound **4b**: ^1^H-NMR (CDCl_3_, 400MHz): δ1.03 (t, J = 6.0 Hz, 6H), 2.56 (q, J = 6.8 Hz, 4H), 2.60 (t, J = 7.6 Hz, 2H), 3.44 (t, J = 4.8 Hz, 2H), 3.92 (s, 3H), 6.97 (d, J = 8.4 Hz, 1H), 7.04 (d, J = 7.6, 1H), 7.89 (s, 1H), 7.99 (d, J = 8.4 Hz, 1H), 8.20, (m, J = 8.4, 1H), 8.46 (s, 1H), 8.84(d, J = 1.6, 1H). ^13^C NMR (CDCl_3_): δ11.8, 37.5, 46.8, 51.4, 55.9, 103.5, 112.3, 117.3, 117.6, 124.3, 129.4, 132.4, 138.2, 142.3, 148.9, 154.5, 158.3, 163.6, 165.1.

#### 4.2.5. Synthesis of ^131^I-N-(4-((2-(diethylamino)ethyl)carbamoyl)-2-iodo-5-methoxyphenyl)- 5-fluoropicolinamide (**5a**) and ^131^I-N-(4-((2-(diethylamino)ethyl)- carbamoyl)-2-iodo-5-methoxyphenyl)-6-fluoronicotinamide (**5b**)

Compound **4a** and **4b** were labeled with radioiodine by the radioiododethallation method to give the desired compounds ^131^I-IFPABZA (**5a**) and ^131^I-IFNABZA (**5b**), respectively. Briefly, either compound **4a** or **4b** (30.0 mg) was dissolved in 0.15 mL of 1.3 mM Tl(TFA)_3_ solution. After incubation at r.t. for 10 min, 15 mL of ^131^I-NaI solution (3 mCi) was added to the solution, and the mixture was reacted at r.t. for another 10 min, and then, evaporated to dryness. The resulting residue was redissolved in dichloromethane and then, loaded to a PLUS silica Sep-Pak, which was sequentially eluted with 5 mL of dichloromethane, 3 mL of MeOH/CH_2_Cl_2_ (1:15, *v/v*), and 5 mL of MeOH/CH_2_Cl_2_ (1:5, *v/v*). The radiolabeling efficiency of each fraction of ^131^I-IFPABZA and ^131^I-IFNABZA crude product was analyzed by radio-thin layer chromatography (radio-TLC) using MeOH/CH_2_Cl_2_ = 1/10 (*v*/*v*) and 1/8 (*v/v*) as the developing phase, respectively (Figure 2). The collected fractions were evaporated to dryness, redissolved in 10% ethanol in normal saline (*v/v*), and filtered by 0.22 mm filter to give the final product.

### 4.3. Partition Coefficient of ^131^I-IFPABZA and ^131^I-IFNABZA

The partition coefficient of ^131^I-IFPABZA and ^131^I-IFNABZA was assessed by determining their distribution between 1-octanol and PBS. Approximately 7.4 kBq of ^131^I-IFPABZA or ^131^I-IFNABZA was added to a tube containing 1 mL of 1-octanol and 1 mL of PBS. The tube was vortexed for 5 min and centrifuged at 3000× *g* for 5 min. Aliquots (0.5 mL) of 1-octanol were taken and added into the next tube containing 0.5 mL of 1-octanol and 1 mL of fresh PBS. These procedures were repeated 5 times. Finally, counting samples (100 µL) of each layer were aspirated and the radioactivity was counted by a gamma counter. The partition coefficient was expressed as log *P*, which was calculated as follows.

(1) $$\mathrm{Log}\;P=\mathrm{Log}\;\frac{activity\;in\;1-Octanol}{activity\;in\;PBS}$$

### 4.4. In Vitro Stability of ^131^I-IFPABZA and ^131^I-IFNABZA

Purified ^131^I-IFPABZA and ^131^I-IFNABZA were incubated either in 1 mL of PBS at 4 °C or in FBS at 37 °C. The percentage of intact radioactive compound, determined by radio-TLC, in different incubation conditions, was considered as an index of in vitro stability at designated time points (1, 2, 4, 24, and 48 h).

### 4.5. Binding Affinity to Melanin

To assess the affinity of ^131^I-IFPABZA and ^131^I-IFNABZA to melanin, 10 mCi of ^131^I-IFPABZA and ^131^I-IFNABZA was incubated with various concentrations of melanin solution (0~200 ppm) at 37 °C for 1 h. After incubation, the mixture was centrifuged at 20,000× *g* for 5 min, and the supernatant was filtered by a 0.22 mm membrane filter. The radioactivity of the pellet and the filtrate was measured by the g-counter. To investigate the time effect on the binding affinity to melanin, both ^131^I-IFPABZA and ^131^I-IFNABZA (10 mCi) were incubated with 50 ppm of melanin solution at 37 °C for 0.5, 1, 1.5, and 2 h. The binding affinity was calculated as follows.

(2) $$\%\;\mathrm{radiotracer}\;\mathrm{bound}=1-\frac{radioactivity\;in\;filtrate\;}{total\;radioactivity}$$

### 4.6. Cell Cultures and Xenograft Inoculation

The B16F10 murine melanoma cells and A375 human amelanotic melanoma cells were grown in Dulbecco’s modified Eagle high-glucose medium (Gibco Life Sciences) supplemented with 10% FBS in a humidified atmosphere containing 5% CO_2_. To conduct subcutaneous tumor inoculation, 5 × 10^5^ of B16F10 and 1 × 10^6^ of A375 cells in 100 µL of serum-free medium were implanted into the right flank of 6-week-old male C57BL/6 mouse and male BALB/c nude mouse, respectively. When the tumor reached 150 ± 50 mm^3^, the mice were selected for the following biological studies. All animal studies were approved by the Institutional Animal Care and Use Committee (IACUC) of National Yang-Ming University (No. 1060604).

### 4.7. In Vitro Cellular Uptake Assay

Cells were seeded in a 6-well plate at a density of 1 × 10^6^ cells/well and cultured in 3 mL of culture medium containing 10% FBS at 37 °C overnight. The medium was replaced by fresh medium containing ^131^I-IFPABZA or ^131^I-IFNABZA (3 mL, 1 µCi/mL). At 15, 30, 60, 120, 240, and 480 min post incubation at 37 °C, the medium was aspirated, and cells were washed twice with 0.5 mL of PBS. The medium and washing PBS were collected in a counting tube. Cells were treated with 0.5 mL of 0.25% trypsin for 5 min for detachment. The cells were harvested and the well was washed twice with 0.5 mL of PBS. The suspension and washing PBS were collected in an anther counting tube and the number of viable cells was determined using hemocytometry. Radioactivity was measured by the g-counter and the cellular uptake of ^131^I-IFNABZA and ^131^I-IFPABZA was expressed as the percentage of administrated dose per one million cells (%AD/10^6^ cells).

### 4.8. Scintigraphic Imaging

The dual head gamma camera (e. cam Signature, Siemens, Germany) equipped with a 4-mm pinhole collimator was applied for the scintigraphic imaging. Both melanotic B16F10 melanoma and amelanotic A375 melanoma-bearing mouse models (n = 3 in each group) were treated with Lugol’s solution before imaging. The scintigraphic imaging was conducted 1, 4, 24, 48, and 96 h after intravenous injection of 200 mCi of ^131^I-IFPABZA or ^131^I-IFNABZA. During imaging, mice were anesthetized with 1–3% isoflurane in 2L of oxygen in the prone position. For quantitative data analysis, a region of interest (ROI) was placed on the tumor and contralateral side of muscle. The tumor-to-muscle ratios (*T/M*) were calculated on the basis of counts per pixel in the ROIs.

### 4.9. Biodistribution Study

The biodistribution of ^131^I-IFNABZA in both B16F10 melanoma- and A375 amelanotic melanoma-bearing mice was conducted. Mice were treated with Lugol’s solution before receiving an injection of ^131^I-IFNABZA (20 mCi/mice). Four mice in each group were sacrificed at 5, 15 min, 1, 4, 24, 48, and 96 h post injection of ^131^I-IFNABZA. The tissues/organs were harvested, weighted, and subjected to radioactivity measurement using the g-counter. The results were expressed as percentage of injected dose per gram of tissue/organ (%ID/g).

### 4.10. Calculation of the Estimated Absorption Dose of Normal Organs

For estimating the radiation absorption dose of various tissues/organs in the human body, we followed the methods reported in our previous studies [16,30]. The radioactive compound and its radioactive metabolites were assumed to distribute homogeneously in tissues/organs, and the relative tissue/organ weight was calculated based on previous studies [31,32]. Based on the results of the biodistribution study in mice, the calculated mean value of the percentage of injected activity per gram of tissue/organ (%IA/g) was extrapolated to the uptake in organs of a 73 kg adult using the following formula.

(3) [(%IA/gorgan)mice×(kgtotal body weight)mice]×(gorgan/(Kgtotal body weight)human)=(%IA/organ)human

The extrapolated values (%IA) in the human organs at 5, 15 min, 1, 4, 24, 48, and 96 h were exponentially fitted and integrated to obtain the number of disintegrations in the source organs. The value of each source organ was input into the OLINDA/EXM 1.0 computer program for the dosimetry estimation.

## 5. Conclusions

Two novel benzamide derivatives, radioiodinated fluoropicolinamide-benzamide derivative (^131^I-IFPABZA) and fluoronicotinamide-benzamide derivative (^131^I-IFNABZA), have been successfully prepared with acceptable radiochemical yield and high radiochemical purity. The cellular uptake study and scintigraphic imaging revealed that ^131^I-IFNABZA exhibited better melanoma-targeting ability than ^131^I-IFPBZA. A prolonged retention in B16F10 melanoma of ^131^I-IFNABZA was demonstrated in the biodistribution study. The estimated effective dose for the whole body after intravenous injection of ^131^I-IFNABZA was much lower than that of previous studies. Overall, we believe that ^131^I-IFNABZA could be a potential theranostic agent for melanoma.

## Figures and Tables

**Table 1:** Radioactivity biodistribution of B16F10 melanoma-bearing C57BL/6 mice after intravenous injection of 20 μCi of ^131^I-IFNABZA.

Organ	5 min	15 min	1 h	4 h	24 h	48 h	96 h
Blood	1.70 ± 0.15	1.24 ± 0.27	0.75 ± 0.19	0.27 ± 0.04	0.06 ± 0.04	0.01 ± 0.00	0.00 ± 0.00
Heart	7.97 ± 0.30	3.76 ± 1.09	1.69 ± 0.51	0.60 ± 0.15	0.25 ± 0.04	0.17 ± 0.05	0.11 ± 0.03
Lung	23.49 ± 2.20	13.87 ± 2.64	5.50 ± 1.57	2.34 ± 0.96	0.42 ± 0.33	0.09 ± 0.03	0.05 ± 0.01
Liver	11.91 ± 0.93	12.90 ± 0.99	11.12 ± 3.43	4.69 ± 1.66	1.93 ± 0.33	1.59 ± 0.37	0.92 ± 0.11
Stomach	3.60 ± 0.49	3.62 ± 0.78	3.20 ± 1.67	2.11 ± 1.60	0.33 ± 0.13	0.08 ± 0.07	0.03 ± 0.01
Small int.	6.85 ± 2.39	14.27 ± 3.04	9.38 ± 3.10	1.00 ± 0.22	0.24 ± 0.08	0.05 ± 0.03	0.03 ± 0.01
Large int.	4.14 ± 0.39	3.89 ± 0.73	3.10 ± 1.29	2.36 ± 1.16	0.21 ± 0.06	0.04 ± 0.02	0.01 ± 0.01
Spleen	10.09 ± 0.92	11.83 ± 1.53	9.19 ± 1.49	7.32 ± 3.53	3.12 ± 1.28	3.06 ± 1.07	1.16 ± 0.64
Pancreas	9.36 ± 0.55	6.97 ± 1.15	5.26 ± 2.75	1.60 ± 0.48	0.24 ± 0.10	0.10 ± 0.06	0.04 ± 0.01
Bone	3.18 ± 0.19	2.56 ± 0.51	1.43 ± 0.33	0.61 ± 0.22	0.19 ± 0.05	0.15 ± 0.03	0.09 ± 0.04
Muscle	2.89 ± 0.18	1.88 ± 0.34	0.59 ± 0.12	0.15 ± 0.02	0.03 ± 0.01	0.02 ± 0.00	0.01 ± 0.00
Tumor	4.29 ± 0.93	4.90 ± 1.24	5.84 ± 1.80	5.19 ± 2.84	5.06 ± 2.09	5.17 ± 1.53	1.51 ± 0.34
Brain	0.95 ± 0.11	0.65 ± 0.13	0.18 ± 0.04	0.03 ± 0.00	0.00 ± 0.00	0.00 ± 0.00	0.00 ± 0.00
Kidneys	26.80 ± 2.32	23.28 ± 2.78	13.61 ± 3.32	6.33 ± 1.76	1.04 ± 0.25	0.48 ± 0.32	0.21 ± 0.02
Eye ball	7.67 ± 0.84	10.57 ± 1.80	15.57 ± 4.36	17.25 ± 1.11	14.69 ± 3.35	15.45 ± 2.41	14.06 ± 1.93
Urine	2.66 ± 1.56	32.36 ± 25.47	79.37 ± 60.45	20.57 ± 7.97	1.88 ± 1.24	0.15 ± 0.08	0.07 ± 0.02
Feces	1.19 ± 0.24	2.02 ± 0.51	9.14 ± 4.48	244.2 ± 50.65	21.77 ± 11.19	3.35 ± 3.36	0.46 ± 0.16
Bladder	1.78 ± 0.66	2.55 ± 0.34	4.04 ± 2.87	4.21 ± 1.97	0.21 ± 0.16	0.02 ± 0.01	0.01 ± 0.00
**Ratios**							
Tumor/muscle	1.48 ± 0.33	2.61 ± 0.81	9.90 ± 3.66	34.60 ± 19.49	168.7 ± 89.52	258.5 ± 76.50	151.0 ± 34.00
Tumor/blood	2.52 ± 0.59	3.95 ± 1.32	7.79 ± 3.11	19.22 ± 10.90	84.33 ± 66.14	517.0 ± 153.0	N/A
Tumor/liver	0.36 ± 0.08	0.38 ± 0.10	0.53 ± 0.23	1.11 ± 0.72	2.62 ± 1.17	3.25 ± 1.22	1.64 ± 0.42

Results were expressed as the percentage of injected dose per gram of organ/tissue (%ID/g). Each value represented mean ± SD (*n* = 4). Small int., small intestine; large int., large intestine.

**Table 2:** Estimated absorption dose of ^131^I-IFNABZA in a 73 kg adult human.

Organ	Estimated Dose (mSv/MBq^−1^)
Adrenals	2.76 × 10^−2^
Brain	3.88 × 10^−3^
Breasts	1.82 × 10^−2^
Gallbladder Wall	3.33 × 10^−2^
LLI Wall	2.91 × 10^−2^
Small Intestine	2.79 × 10^−2^
Stomach Wall	2.72 × 10^−2^
ULI Wall	2.51 × 10^−2^
Heart Wall	1.48 × 10^−2^
Kidneys	6.27 × 10^−2^
Liver	1.22 × 10^−1^
Lungs	2.25 × 10^−2^
Muscle	1.01 × 10^−2^
Ovaries	2.29 × 10^−2^
Pancreas	2.58 × 10^−2^
Red Marrow	1.80 × 10^−2^
Osteogenic Cells	4.92 × 10^−2^
Skin	1.58 × 10^−2^
Spleen	1.51 × 10^−1^
Testes	1.87 × 10^−2^
Thymus	1.94 × 10^−2^
Thyroid	1.89 × 10^−2^
Urinary Bladder Wall	2.18 × 10^−2^
Uterus	2.33 × 10^−2^
Whole Body	2.37 × 10^−2^
Effective Dose	3.02 × 10^−2^

Radiation-absorbed dosimetry was converted from the biodistribution of ^131^I-IFNABZA in 0.025 kg tumor-bearing mice to a 73 kg adult human.

## Supplementary Materials

The following are available online at https://www.mdpi.com/1422-0067/21/18/6597/s1.
