# Supplementary material for: Evaluation of Radioiodinated Fluoronicotinamide/Fluoropicolinamide-Benzamide Derivatives as Theranostic Agents for Melanoma
Source: Int J Mol Sci. 2020 Sep 9;21(18):6597. doi: 10.3390/ijms21186597 (PMC7554940; doi:10.3390/ijms21186597)
Supplement: Supplementary file 1 [file ijms-21-06597-s001.pdf]

Supplementary Materials:

Table S1. Radioactivity biodistribution of A375 amelanotic melanoma-bearing BALB/c nude mice after intravenous injection of 20 μCi of ^{131}I-IFNABZA.

Organ	5 min	15 min	1h	4h	24h	48h	96h
Blood	1.42 ± 0.35	1.27 ± 0.34	0.85 ± 0.15	0.20 ± 0.04	0.04 ± 0.00	0.02 ± 0.00	0.01 ± 0.01
Heart	7.34 ± 1.68	4.29 ± 0.44	1.26 ± 0.30	0.36 ± 0.10	0.12 ± 0.01	0.08 ± 0.01	0.06 ± 0.01
Lung	24.31 ± 4.86	10.94 ± 1.34	4.54 ± 0.94	2.31 ± 0.48	0.26 ± 0.06	0.09 ± 0.03	0.06 ± 0.01
Liver	14.07 ± 0.54	16.19 ± 0.42	9.75 ± 2.59	3.82 ± 0.64	2.15 ± 0.08	1.51 ± 0.15	0.93 ± 0.18
Stomach	3.33 ± 0.23	3.62 ± 0.33	3.49 ± 2.35	0.70 ± 0.23	0.15 ± 0.02	0.06 ± 0.01	0.03 ± 0.01
Small int.	5.70 ± 1.01	8.09 ± 0.91	4.47 ± 1.48	0.56 ± 0.05	0.09 ± 0.02	0.04 ± 0.01	0.02 ± 0.01
Large int.	3.93 ± 0.89	3.39 ± 0.45	2.10 ± 0.57	1.37 ± 0.42	0.07 ± 0.02	0.02 ± 0.01	0.01 ± 0.01
Spleen	6.77 ± 1.57	8.59 ± 0.82	7.06 ± 1.31	3.13 ± 0.59	1.86 ± 0.41	1.16 ± 0.19	0.75 ± 0.14
Pancreas	8.62 ± 1.38	7.08 ± 0.19	3.72 ± 0.99	1.56 ± 0.52	0.31 ± 0.08	0.11 ± 0.01	0.07 ± 0.01
Bone	1.74 ± 0.17	1.84 ± 0.02	0.98 ± 0.19	0.36 ± 0.08	0.09 ± 0.01	0.04 ± 0.01	0.02 ± 0.01
Muscle	2.20 ± 0.21	1.48 ± 0.37	0.69 ± 0.23	0.12 ± 0.03	0.03 ± 0.00	0.01 ± 0.01	0.01 ± 0.00
Tumor	1.66 ± 0.39	1.68 ± 0.23	1.73 ± 0.51	0.34 ± 0.07	0.10 ± 0.05	0.05 ± 0.02	0.02 ± 0.01
Brain	0.71 ± 0.16	0.48 ± 0.01	0.17 ± 0.04	0.02 ± 0.00	0.00 ± 0.00	0.00 ± 0.00	0.00 ± 0.00
Kidneys	27.07 ± 3.54	25.15 ± 1.97	12.06 ± 3.05	5.13 ± 1.52	0.99 ± 0.15	0.37 ± 0.02	0.19 ± 0.01
Eye ball	1.20 ± 0.26	1.11 ± 0.16	0.80 ± 0.19	0.28 ± 0.09	0.04 ± 0.01	0.01 ± 0.01	0.00 ± 0.00
Urine	2.44 ± 0.18	42.30 ± 34.83	175.9 ± 94.4	20.39 ± 9.71	0.57 ± 0.09	0.14 ± 0.08	0.05 ± 0.02
Feces	1.12 ± 0.28	2.64 ± 0.89	15.96 ± 5.57	73.25 ± 26.98	6.23 ± 4.04	0.82 ± 0.20	0.20 ± 0.05
Bladder	1.69 ± 0.37	2.11 ± 0.67	1.56 ± 0.79	0.42 ± 0.25	0.04 ± 0.02	0.02 ± 0.01	0.00 ± 0.00
Uptake ratio							
Tumor/muscle	0.75 ± 0.19	1.14 ± 0.32	2.04 ± 0.70	1.70 ± 0.49	2.50 ± 1.25	2.50 ± 1.00	2.00 ± 1.00
Tumor/blood	1.17 ± 0.40	1.32 ± 0.40	2.51 ± 1.12	2.83 ± 0.92	3.33 ± 2.00	5.00 ± 5.39	N/A
Tumor/liver	0.12 ± 0.03	0.10 ± 0.01	0.85 ± 0.15	0.20 ± 0.04	0.04 ± 0.00	0.02 ± 0.00	0.01 ± 0.01

Results were expressed as the percentage of injected dose per gram of organ/tissue (%ID/g). Each value represented mean ± SD (*n* = 4). Small int., small intestine; large int., large intestine.

Table S2. Radioactivity biodistribution of healthy BALB/c mice at 5 min post administration of 20 mCi of ^{131}I-IFNABZA dissolved in different excipients.

Organ	Excipient A			Excipient B			Excipient C		
Blood	1.34	±	0.40	1.08	±	0.12	1.10	±	0.07
Lung	14.28	±	1.86	16.00	±	1.64	15.11	±	2.11
Liver	16.32	±	2.19	16.26	±	1.50	20.18	±	1.45
Kidneys	25.47	±	1.16	26.21	±	2.08	28.59	±	1.90
Muscle	1.84	±	0.21	1.99	±	0.19	1.87	±	0.31

Excipient A: 10% ethanol in normal saline
Excipient B: 5% Tween 80 and 5% ethanol in normal saline
Excipient C: 1% bovine serum albumin and 5% ethanol in normal saline

Results were expressed as the percentage of injected dose per gram of organ/tissue (%ID/g).Each value represented mean ± SD (n = 5).

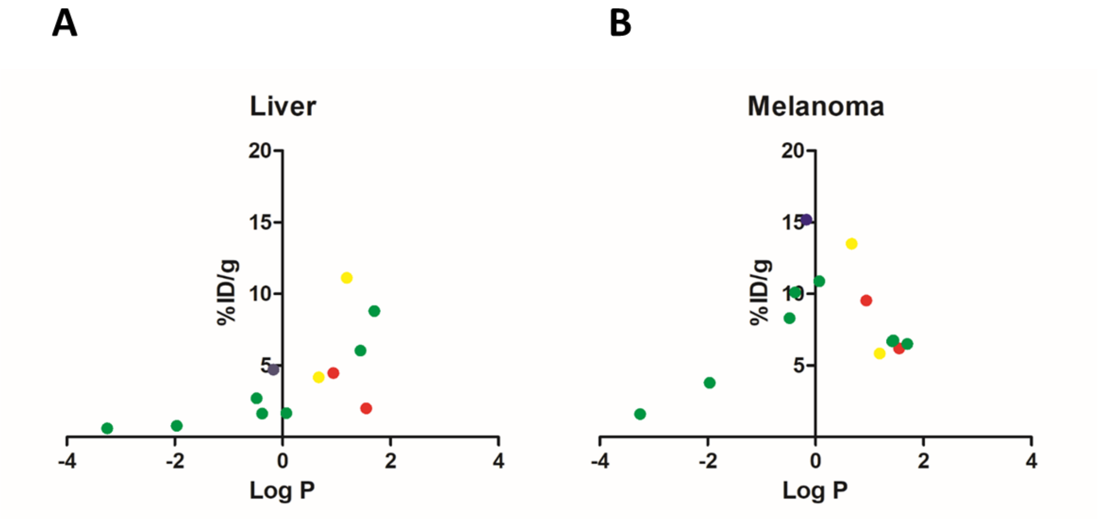

Category	Compound	Log *P*	Reference
Benzamide derivatives	^{125}I-IBZA	1.44	[1]
	^{123}I-IMBA	1.42	[6]
	^{131}I-IHPA	-0.38	[7]
	^{131}I-IHPP	0.07	[7]
	^{18}F-FBZA	1.70	[10]
	^{18}F-FPBZA	-0.48	[4]
	Al^{18}F-NOTA-BZA	-1.96	[5]
	^{68}GA-SCN-NOTA-BZA	-3.25	[9]
Nicotinamide derivatives	^{123}I-MEL008	1.55	[8]
	^{18}F-MEL050	0.94	
Picolinamide derivative	^{18}F-FPN	-0.17	[2]
Nicotinamide-benzamide derivatives	^{131}I-ICNA	0.67	[3]
	^{131}I-IFNABZA	1.19	

Figure S1. The relationship between lipophilicity and radioactivity accumulation of radiolabeled ligands in (**A**) liver and (**B**) melanoma. The data were collected based on previous literature. Green dots: Benzamide derivatives [1,4–7,9,10]; red dots: Nicotinamide derivatives [8]; black dots: Picolinamide derivative [2]; yellow dots: Nicotinamide-benzamide derivatives [3]

Reference

1. Michelot, J.M.; Moreau, M.F.; Veyre, A.J.; Bonafous, J.F.; Bacin, F.J.; Madelmont, J.C.; Bussiere, F.; Souteyrand, P.A.; Mauclaire, L.P.; Chossat, F.M.; et al. Phase II scintigraphic clinical trial of malignant melanoma and metastases with iodine-123-N-(2-diethylaminoethyl 4-iodobenzamide). *J. Nucl. Med.* **1993**, *34*, 1260–1266.
2. Greguric, I.; Taylor, S.R.; Denoyer, D.; Ballantyne, P.; Berghofer, P.; Roselt, P.; Pham, T.Q.; Mattner, F.; Bourdier, T.; Neels, O.C.; et al. Discovery of [^{18}F]N-(2-(diethylamino)ethyl)-6-fluoronicotinamide: A melanoma positron emission tomography imaging radiotracer with high tumor to body contrast ratio and rapid renal clearance. *J. Med. Chem.* **2009**, *52*, 5299–5302.
3. Chang, C.C.; Chang, C.H.; Shen, C.C.; Chen, C.L.; Liu, R.S.; Lin, M.H.; Wang, H.E. Synthesis and evaluation of $^{123/131}$I-Iochlonicotinamide as a novel SPECT probe for malignant melanoma. *Bioorg. Med. Chem.* **2015**, *23*, 2261–2269.
4. Wu, S.Y.; Huang, S.P.; Lo, Y.C.; Liu, R.S.; Wang, S.J.; Lin, W.J.; Shen, C.C.; Wang, H.E. Synthesis and preclinical characterization of [^{18}F]FPBZA: A novel PET probe for melanoma. *Biomed. Res. Int.* **2014**, *2014*, 912498, doi:10.1155/2014/912498.
5. Chang, C.C.; Chang, C.H.; Lo, Y.H.; Lin, M.H.; Shen, C.C.; Liu, R.S.; Wang, H.E.; Chen, C.L. Preparation and characterization of a novel Al^{18}F-NOTA-BZA conjugate for melanin-targeted imaging of malignant melanoma. *Bioorg. Med. Chem. Lett.* **2016**, *26*, 4133–4139.
6. Nicholl, C.; Mohammed, A.; Hull, W.E.; Bubeck, B.; Eisenhut, M. Pharmacokinetics of iodine-123-IMBA for melanoma imaging. *J. Nucl. Med.* **1997**, *38*, 127–133.
7. Chang, C.C.; Chang, C.H.; Shen, C.C.; Chen, C.L.; Liu, R.S.; Lin, M.H.; Wang, H.E. Synthesis and characterization of a novel radioiodinated phenylacetamide and its homolog as theranostic agents for malignant melanoma. *Eur. J. Pharm. Sci.* **2016**, *81*, 201–209.
8. Chezal, J.M.; Papon, J.; Labarre, P.; Lartigue, C.; Galmier, M.J.; Decombat, C.; Chavignon, O.; Maublant, J.; Teulade, J.C.; Madelmont, J.C.; et al. Evaluation of radiolabeled (hetero)aromatic analogues of N-(2-diethylaminoethyl)-4-iodobenzamide for imaging and targeted radionuclide therapy of melanoma. *J. Med. Chem.* **2008**, *51*, 3133–3144.
9. Kim, H.J.; Kim, D.Y.; Park, J.H.; Yang, S.D.; Hur, M.G.; Min, J.J.; Yu, K.H. Synthesis and characterization of a ^{68}Ga-labeled N-(2-diethylaminoethyl)benzamide derivative as potential PET probe for malignant melanoma. *Bioorg. Med. Chem.* **2012**, *20*, 4915–4920.
10. Ren, G.; Miao, Z.; Liu, H.; Jiang, L.; Limpa-Amara, N.; Mahmood, A.; Gambhir, S.S.; Cheng, Z. Melanin-targeted preclinical PET imaging of melanoma metastasis. *J. Nucl. Med.* **2009**, *50*, 1692–1699.
